# Supplementary material for: Genetic comparisons of interleukin-17 reveal a framework for complex signaling evolution
Source: bioRxiv. 2026 Apr 14:2026.04.13.718218. Preprint. [Version 1] doi: 10.64898/2026.04.13.718218 (PMC13105070; doi:10.64898/2026.04.13.718218)
Supplement: Supplement 3 [file media-3.zip › Supplemental files for paper/ERC/IL17xIL17_comparisons/IL17C_IL17RB.pdf]

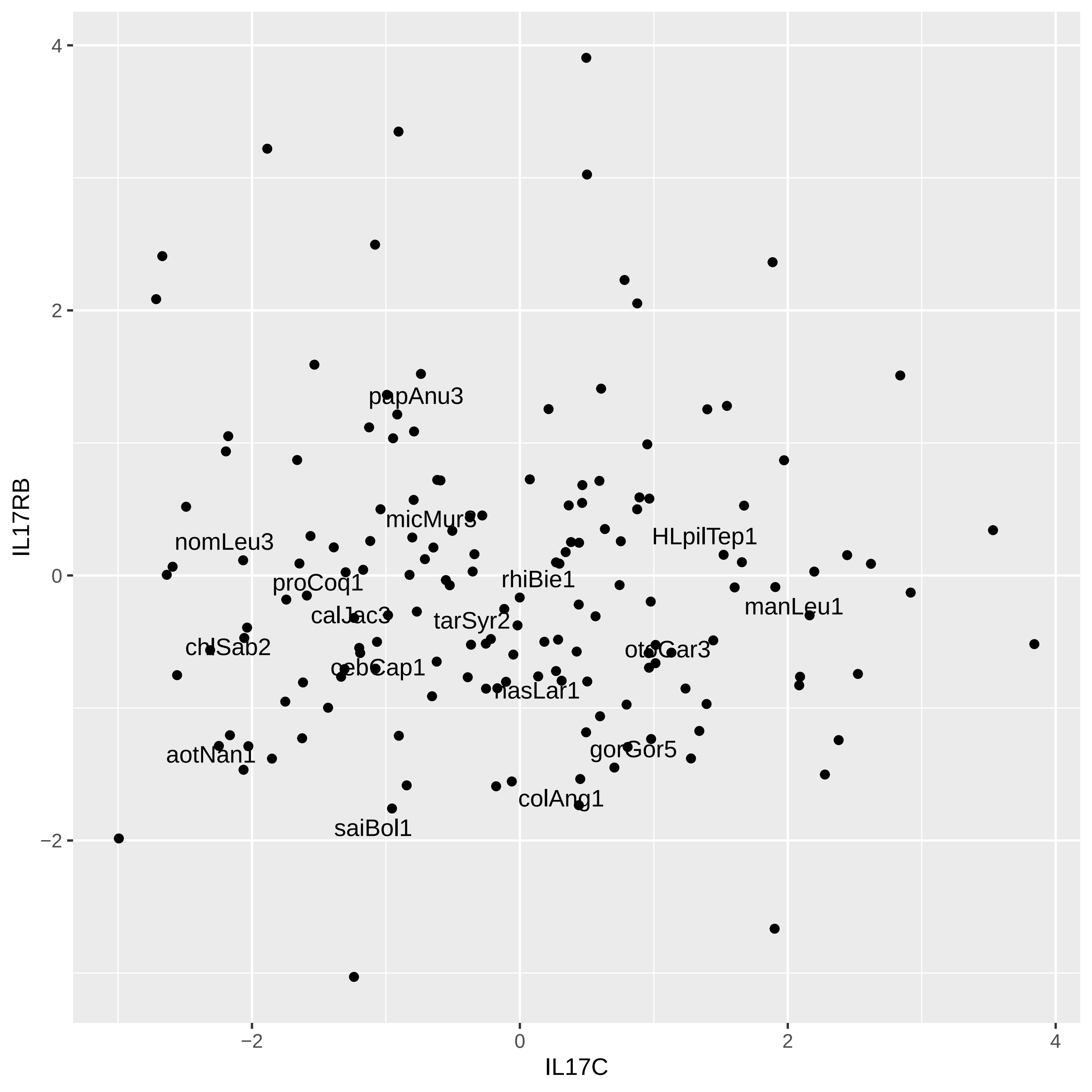

IL17RB
IL17C
4
2
0
−2
−2
0
2
4
papAnu3
micMur3
nomLeu3
HLpilTep1
proCoq1
rhiBie1
manLeu1
calJac3
tarSyr2
chlSab2
otoGar3
cebCap1
nasLar1
aotNan1
gorGor5
colAng1
saiBol1
